# Novel Complex of PD-L1 Aptamer and Holliday Junction Enhances Antitumor Efficacy in Vivo

**DOI:** 10.3390/molecules26041067

**Published:** 2021-02-18

**Authors:** Ting Li, Fengjiao Yao, Yacong An, Xundou Li, Jinhong Duan, Xian-Da Yang

**Affiliations:** Institute of Basic Medical Sciences, Chinese Academy of Medical Sciences & Peking Union Medical College, Beijing 100005, China; Tingli2018@163.com (T.L.); fjyao_1103@126.com (F.Y.); anyacong@ibms.pumc.edu.cn (Y.A.); lixd1012@163.com (X.L.); jinhong_duan@aliyun.com (J.D.)

**Keywords:** aptamer, Holliday Junction, immunotherapy

## Abstract

Blocking the PD-1/PD-L1 pathway can diminish immunosuppression and enhance anticancer immunity. PD-1/PD-L1 blockade can be realized by aptamers, which have good biocompatibility and can be synthesized in quantity economically. For in vivo applications, aptamers need to evade renal clearance and nuclease digestion. Here we investigated whether DNA nanostructures could be used to enhance the function of PD-L1 aptamers. Four PD-L1 aptamers (Apt) were built into a Holliday Junction (HJ) to form a tetravalent DNA nanostructure (Apt-HJ). The average size of Apt-HJ was 13.22 nm, which was above the threshold for renal clearance. Apt-HJ also underwent partial phosphorothioate modification and had improved nuclease resistance. Compared with the monovalent PD-L1 aptamer, the tetravalent Apt-HJ had stronger affinity to CT26 colon cancer cells. Moreover, Apt-HJ markedly boosted the antitumor efficacy in vivo vs. free PD-L1 aptamers without raising systemic toxicity. The results indicate that multiple aptamers attached to a DNA nanostructure may significantly improve the function of PD-L1 aptamers in vivo.

## 1. Introduction

Cancer is a leading cause of mortality in the 21st century. There are four main treatments for cancer: surgery, radiotherapy, chemotherapy, and immunotherapy. Unlike the first three treatments that directly target the cancer cells, immunotherapy attacks the tumor through mobilization of the host′s immune system [1]. In recent years, cancer immunotherapy has become an area of focus of medical oncology. One promising strategy to enhance antitumor immunity is immune checkpoint blockade (ICB), which has opened a new field for modern medicine [2]. In recent years, significant progress has been achieved by ICB therapies, which have shown far greater benefits for patients than previous therapies, bringing hope to patients with advanced malignancies [3]. Among ICB therapies, PD-1/PD-L1 blockade is a mainstream approach and has become a widely used strategy for cancer treatment. PD-1 is expressed on activated T cells and, when combined with its ligand PD-L1, can inhibit the T cell response [4]. PD-L1 is expressed on many types of cells, including e pithelial cells, endothelial cells, and cells comprising the immune system [5]. Above all, PD-L1 is commonly upregulated on the surface of tumor cells [6], and it plays an important role in evading antigen-specific immune responses by suppressing T cell functions [7]. Clinical data have shown that blocking the PD-1/PD-L1 pathway can enhance antitumor immunity, produce a lasting clinical response, and prolong patient survival [8]. Increasing numbers of clinical studies indicate that antibodies targeting PD-1 or PD-L1 can achieve promising results in multiple tumor types [2,9], including melanoma, prostate cancer, and non-small cell lung cancer (NSCLC) [10,11,12]. In melanoma patients treated with PD-1 antibodies, about 20% experience complete response [13]. Moreover, in patients with advanced squamous cell NSCLC, PD-1 antibody can significantly improve overall survival, response rate, and progression-free survival [14]. As a result, the development of PD-1/PD-L1 inhibitors as a form of cancer immunotherapy has gained unprecedented attention.

The current ICB therapies are implemented by antibodies. There are five monoclonal antibodies targeting the PD-1/PD-L1 pathway approved by the FDA: atezolizumab, nivolumab, durvalumab, avelumab, and pembrolizumab [15]. However, repeated use of monoclonal antibodies can be highly immunogenic. This immunogenicity is manifested in the production of anti-drug antibodies (ADAs), which can change the pharmacokinetic and pharmacodynamic properties of the PD-1/PD-L1 inhibitors and reduce their efficacy [16,17]. Moreover, the production of humanized monoclonal antibodies is complex, expensive, and time-consuming [18]. As a result, these ICB therapies are relatively expensive and cannot be afforded by many cancer patients in the world, especially those living in low- and lower-middle-income countries. It is therefore necessary to explore other types of ligands that can also achieve ICB function with a less expensive production process.

An aptamer is a type of ligand with promising advantages for biomedical applications. Aptamers are short single-strand DNA or RNA oligonucleotides, which can fold into complex 3D structures and bind to target molecules with high affinity and specificity [19]. Compared with antibodies, aptamers can be chemically synthesized at relatively low cost and easily modified structurally for biomedical applications [20]. Moreover, aptamers have low immunogenicity and usually do not cause adverse immune effects [21]. What is more, aptamers may have better tissue penetration and faster binding to tumor cells [18]. As the first aptamer-based drug (Macugen, for age-related macular degeneration) was approved by the FDA in 2004 [22], more aptamers for treatment of various diseases have entered clinical trials. These progresses indicate that, similarly to antibodies, aptamers also have the potential to function as specific ligands in clinical applications.

Aptamers have been developed for PD-1/PD-L1 blockade. Lai et al. selected an aptamer for human PD-L1 with relatively high affinity. This PD-L1 aptamer could also bind to recombinant murine PD-L1 protein with a K_d_ of 72nM. As a result, the aptamer could bind to murine PD-L1 expressed on CT26 or LL/2 cells, and generate significant tumor inhibition in both CT26- or LL/2-bearing mice, without observable hepatic or renal toxicity [23]. The results indicate that the PD-L1 aptamer has great potential for future development as an ICB agent.

It should be noted, however, that for in vivo applications, aptamers need to satisfy certain conditions. Aptamers need to have nuclease-resistance, to a certain degree, in order to survive digestion by serum nucleases. Moreover, aptamers generally have a small size and tend to leach out of the kidney [24], which usually does not retain particles less than 10 nm [25]. As a result, most free aptamers are readily excreted into urine through renal filtration, significantly reducing their circulation time within the body. One strategy to circumvent this problem is to attach the aptamer to a DNA nanostructure of a slightly larger size. DNA nanostructures have potential for biomedical applications due to advantages of good biocompatibility, low cytotoxicity, and self-assembly capability through Watson–Crick base-pairing [26,27]. A Holliday Junction (HJ) is a popular DNA nanostructure that can serve as a carrier to deliver drugs, nucleic acids, and enzymes [26]. It has the shape of a cross and is composed of four single-stranded DNA chains. The size of HJs is relatively small, but larger than that of a free aptamer, preventing HJs from leaking out via renal clearance. Moreover, the size of HJs is also appropriate for enrichment in tumor tissue via the enhanced permeability and retention (EPR) effect, because tumor blood vessels are usually more permeable, allowing proper-sized nanoparticles to accumulate around the tumor. Another important advantage of HJs is their cross shape with four arms, which can be connected to four aptamers to form a tetravalent structure, thereby possibly increasing their affinity for target cancer cells.

To date, no report in the literature has evaluated the application of a DNA nanostructure to improve the efficacy of PD-1/PD-L1 blockade by aptamers. In this study, four PD-L1 aptamers were built into the four arms of HJ to form Apt-HJ. Compared with free PD-L1 aptamers, we found that the tetravalent Apt-HJ had stronger affinity to CT26 cells and significantly improved antitumor efficacy in vivo.

## 2. Results

### 2.1. Preparation and Characterization of Apt-HJ

**Figure 1:**
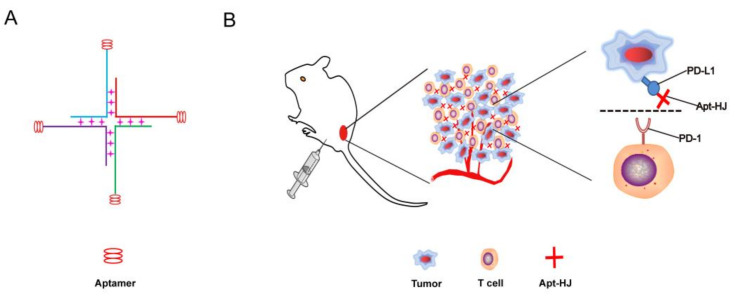
Schematic description of an aptamer Holliday Junction (Apt-HJ) designed for colon cancer treatment. (**A**) The structure of Apt-HJ, which was made of four ssDNA chains. Each ssDNA had a PD-L1 aptamer at the 3′ end and the sequence for HJ at the 5′ end. (**B**) Schematic illustration of Apt-HJ’s binding to PD-L1 on the surface of tumor cells for blockade of PD-L1/PD-1 interaction.

**Figure 2:**
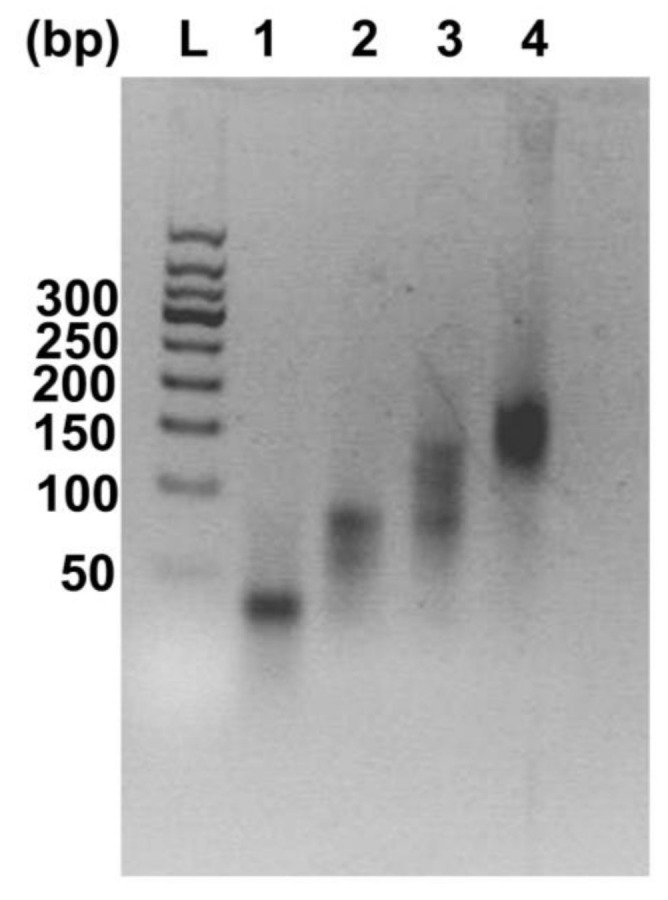
Agarose gel electrophoresis of the DNAs used for the formation of DNA nanostructures. Lane L is a 50 bp DNA ladder. Lanes 1–4 represent images of DNA mixtures containing 1, 2, 3, and 4 ssDNA chains, respectively.

Previous studies have shown that a PD-L1 aptamer can generate an anti-tumor effect in vivo [23]. In this study, we designed a DNA nanostructure that had four PD-L1 aptamers built into it. The nanostructure was made of four ssDNA chains. Each ssDNA had a PD-L1 aptamer at the 3′ end, and the sequence for Holliday Junction at the 5′ end, with a 5 nt poly-A linker in between. Theoretically, the four ssDNA chains could form a cross-shaped tetravalent DNA nanostructure (Apt-HJ) through self-assembly via DNA base pairing (Figure 1). To evaluate whether the four ssDNA chains indeed formed Apt-HJ, agarose gel electrophoresis was performed. As shown in Figure 2, as the DNA valence increased, larger structures were formed. When all four ssDNAs were mixed together, a band of largest molecular weight was observed, indicating that the tetravalent Apt-HJ was formed as expected.

**Figure 3:**
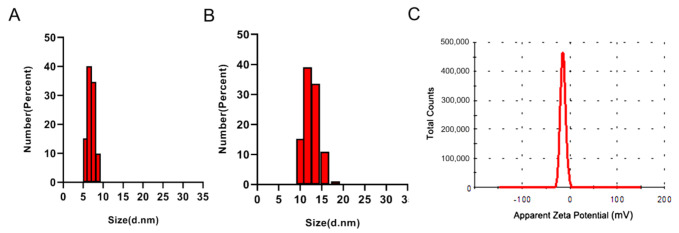
Characterization of PD-L1 aptamers and Apt-HJ by dynamic light scattering (DLS). (**A**) Size distributions of free PD-L1 aptamers. (**B**) Size distribution of Apt-HJ. (**C**) Zeta potential distribution of Apt-HJ.

It is well known that the threshold for kidneys to clear nanoparticles is approximately 10 nm [25]. To evaluate the size of the DNA nanostructures, a dynamic light scattering (DLS) study was conducted. As shown in Figure 3, the average hydrodynamic diameter of an Apt-HJ was 13.22 nm, whereas the size of a free PD-L1 aptamer was 7.14 nm. The results suggested that Apt-HJs had a size above the renal clearance threshold, whereas free aptamers had a size below the threshold. Of note, similar sized particles usually will not be phagocytized by the reticuloendothelial system (RES) after systemic administration [28]. The zeta-potential of Apt-HJ was also measured, with an average value of −11.9 mV (Figure 3C).

### 2.2. Serum Stability of Apt-HJ

**Figure 4:**
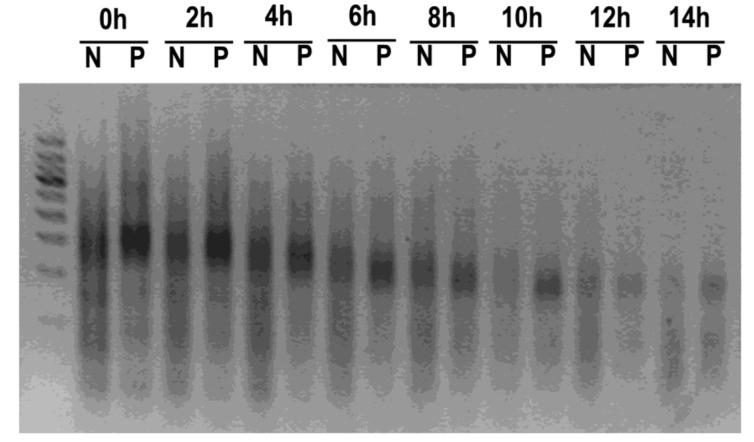
Electrophoresis of Apt-HJ after incubation in 50% serum for various time periods. (N: unmodified Apt-HJ; P: phosphorothioate-modified Apt-HJ).

A main obstacle for in vivo application of Apt-HJ is digestion by nucleases, due to the presence of large amounts of nucleases in serum. Various chemical methods have been developed to slow down the digestion of DNA strands by nucleases [29,30]. Phosphorothioate modification of the DNA backbone has been shown to protect DNA from degradation reproducibly [31,32]. In this study, the first nucleotide at the 5′ end of each ssDNA for Apt-HJ was modified with phosphorothioate. To evaluate whether this modification could improve nuclease resistance, phosphorothioate-modified Apt-HJ and unmodified Apt-HJ were incubated with 50% serum for various time periods and analyzed by electrophoresis. In the presence of serum, phosphorothioate-modified Apt-HJ lasted longer than unmodified Apt-HJ (Figure 4). The results indicated that phosphorothioate modification could significantly improve nuclease resistance and serum stability, and possibly prolong the circulation time of Apt-HJ. Subsequent experiments were conducted using phosphorothioate-modified Apt-HJ.

### 2.3. Affinity of Apt-HJ to Target Cancer Cells

**Figure 5:**
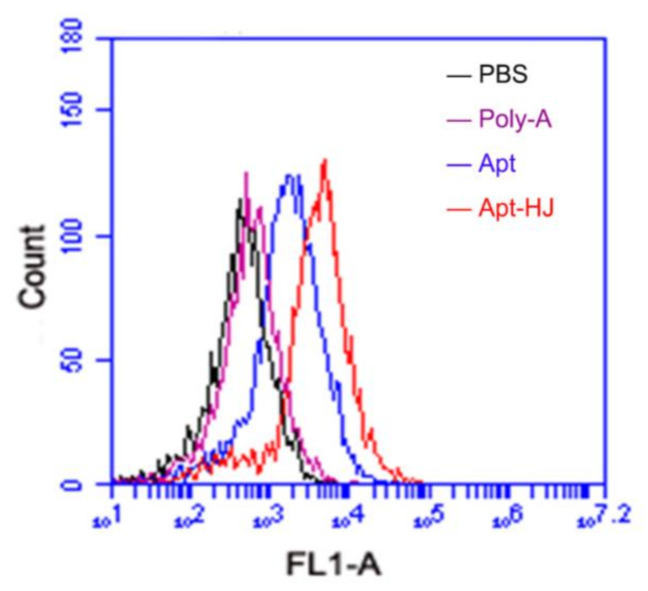
Flow cytometry analysis of CT26 cells after incubation with PBS (black), Carboxyfluorescein (FAM)-labeled poly-A (purple), PD-L1 Apt (blue), or Apt-HJ (red). CT26 cells (2 × 10^5^) were treated with FAM-labeled poly-A, Apt, or Apt-HJ (60 pmol/250 μL), washed thrice with PBS, and analyzed by flow cytometry.

**Figure 6:**
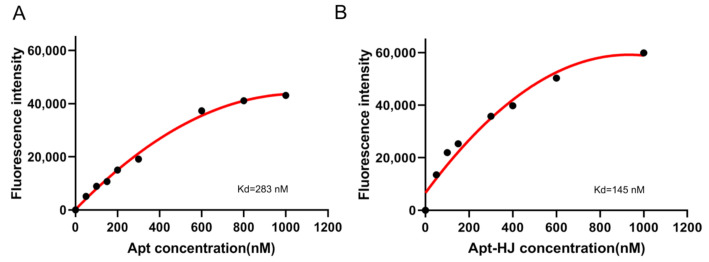
Estimation of apparent binding affinity to CT26 cells by free PD-L1 Apt (**A**) or Apt-HJ (**B**). CT26 cells were incubated with FAM-labeled PD-L1 aptamers or Apt-HJ of various concentrations for 30 min, washed thrice in PBS, and analyzed by flow cytometry. Duplicate samples (*n* = 2) were used for each concentration in the experiment. The mean of the two fluorescence measurements was used in the chart.

**Figure 7:**
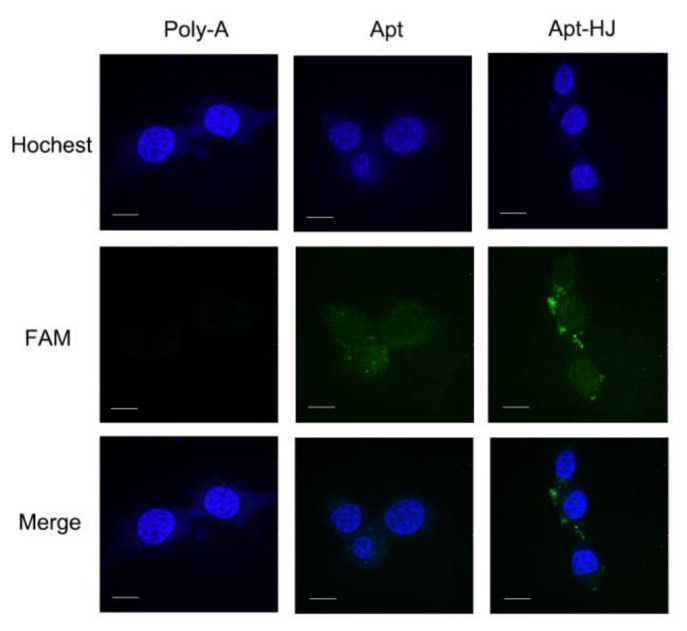
Confocal microscopic images of CT26 cells treated with FAM-labeled poly-A DNA, PD-L1 Apt, or Apt-HJ. The cell nuclei were stained blue with Hoechst. Scale bar: 10 μm.

It has been reported that CT26 murine colon cancer cells express PD-L1, and that the PD-L1 aptamers can bind with these cells and act as an ICB agent [23]. Since the PD-L1 aptamers in Apt-HJ were attached to the Holliday Junction and might have structural change, it was unknown whether Apt-HJ would retain the capability to bind with CT26 cells. To evaluate the targeting affinity of Apt-HJ vs. free PD-L1 aptamers, flow cytometry analysis was conducted. For the sake of fair comparison, each Apt-HJ was labeled with one FAM molecule, as was each free PD-L1 aptamer. CT26 colon cancer cells were incubated with equimolar FAM-labeled poly-A sequences (control), free PD-L1 aptamers, or Apt-HJ. As shown in Figure 5, poly-A did not bind to CT26 cells, free PD-L1 aptamers generated some fluorescence, and Apt-HJ generated by far the strongest fluorescence. The results indicated that both free PD-L1 aptamers and Apt-HJ could bind to target cancer cells, with the latter having the higher affinity, presumably because Apt-HJ was tetravalent and had a stronger attachment vs. the monovalent free aptamers. To quantitatively evaluate the apparent binding affinity of free aptamers or Apt-HJ to CT26 cells, the K_d_ values were also measured and calculated (Figure 6). The K_d_ of free aptamer for CT26 cells was estimated to be 283 nM, while that of Apt-HJ was 145 nM. The results again indicated that Apt-HJ had a higher apparent binding affinity to CT26 cells vs. the free aptamers. To further confirm that PD-L1 aptamers and Apt-HJ could bind to CT26 cancer cells, equimolar FAM-labeled poly-A DNA sequences (control), free PD-L1 aptamers, or Apt-HJ were incubated with CT26 cells, which were subsequently fixed and imaged by confocal microscopy. As shown in Figure 7, poly-A generated almost no signal, free PD-L1 aptamers generated some fluorescence, while Apt-HJ generated the strongest green fluorescence. The results again suggested that Apt-HJ could bind to CT26 cells more tightly than free PD-L1 aptamers.

### 2.4. In Vivo Antitumor Study

**Figure 8:**
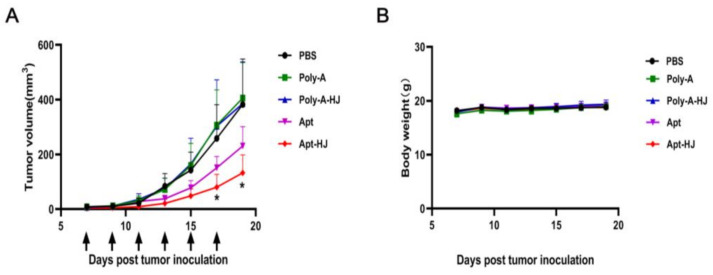
In vivo antitumor study with CT26-bearing BALB/c mice (*n* = 7). CT26 tumor-bearing mice were treated with PBS, poly-A (control DNA sequence), poly-A-HJ, free PD-L1 aptamers, or Apt-HJ. The treatments were administered via intraperitoneal injection every two days, for a total of six injections (arrows). (**A**) Tumor volume and (**B**) body weights were recorded for various treatment groups. Stars indicate statistically significant differences (*p* < 0.05) between the Apt and the Apt-HJ groups, with *p* values of 0.011 and 0.020, respectively (raw data are presented in Table S1).

Although Apt-HJ had superior target affinity vs. free aptamers in vitro, it was still unclear whether Apt-HJ would generate antitumor efficacy in vivo. To address this issue, CT26 tumor-bearing mice were treated with PBS, poly-A (control DNA sequence), poly-A-HJ, free PD-L1 aptamers, or Apt-HJ. The treatments were given via intraperitoneal injection every two days, for a total of six injections. The dosage of free PD-L1 aptamers was 1.2 mg/kg. Poly-A treatment had the same molar concentration as free PD-L1 aptamers. Since Apt-HJ and poly-A-HJ were tetravalent, they were given a molar concentration that was 1/4 of the free PD-L1 aptamers, for the sake of fair comparison. As shown in Figure 8A, although free PD-L1 aptamers generated tumor inhibition to a certain degree, significantly stronger antitumor efficacy was observed in the Apt-HJ treatment group. Poly-A or poly-A-HJ had no obvious antitumor effects, suggesting that the antitumor effect was not due to HJ. To evaluate systemic toxicity, the body weights of animals were also recorded, with no difference detected among the treatment groups (Figure 8B). The results indicated that Apt-HJ enhanced the antitumor efficacy of the PD-L1 aptamers in vivo, but it did not generate additional systemic toxicity.

## 3. Discussion

In this study, a new DNA nanostructure (Apt-HJ) comprising four PD-L1 aptamers and a Holliday Junction was constructed to enhance the target affinity and the in vivo antitumor efficacy. Apt-HJ was made of four ssDNA chains which had the PD-L1 aptamer at their 3′ end and the HJ sequence at their 5′ end. These ssDNA chains were assembled into a tetravalent structure via a self-assembly process (Figure 1 and Figure 2). The average diameter of the Apt-HJ was about 13.22 nm (Figure 3). Partial phosphorothioate modification of Apt-HJ enhanced its nuclease resistance and serum stability (Figure 4). Compared with the monovalent free PD-L1 aptamer, the tetravalent Apt-HJ exhibited higher affinity to CT26 colon cancer cells (Figure 5, Figure 6 and Figure 7). Moreover, Apt-HJ significantly improved the anticancer efficacy in tumor-bearing mice vs. free PD-L1 aptamers (Figure 8), indicating that Apt-HJ might be better suited for in vivo ICB therapies.

A major obstacle for in vivo applications of aptamers is their rapid renal clearance and short circulating time. To address this issue, aptamers are sometimes conjugated to carriers with a high molecular mass to increase their size [33]. PEGylation is the most commonly used method to prevent renal leakage of aptamers. However, PEGylation has some issues that cannot be completely ignored. Because PEG can induce immune response and generate antibodies, a PEG-modified drug may bind to the antibody and be eliminated by phagocytes that express the Fc receptors, resulting in accelerated blood clearance and a significantly shortened half-life [34]. Moreover, due to PEG’s non-biodegradability, its components may induce unforeseeable biological effects. Allergic reactions to PEGylated formulations have been reported [35,36,37]. Furthermore, since PEG is covalently conjugated to aptamers, chemical reactions are required in the drug production process. In this study, a DNA nanostructure was employed to evade renal clearance, with several advantages. The DNA nanostructure of Apt-HJ can be formed through self-assembly, which is a relatively simple and cost-efficient process. Moreover, unlike PEG, DNA nanostructure is biodegradable and has good biocompatibility with low immunogenicity. Another prerequisite for in vivo aptamer application is nuclease resistance. In this study, the DNA nanostructure was strengthened by partial phosphorothioate modification, which markedly improved the nuclease resistance of Apt-HJ (Figure 4). Each strand of DNA that composes Apt-HJ has 85 nt (including the PD-L1 aptamer, a 5 nt poly-A spacer, and the HJ sequence), which can be synthesized economically. Taken together, attaching aptamers to a DNA nanostructure may be another strategy to evade renal clearance in vivo.

**Figure 9:**
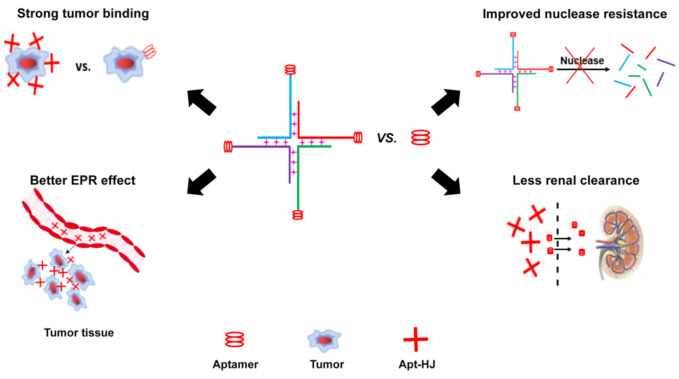
Schematic illustration of the possible working mechanisms of Apt-HJ. Compared with free Apt, Apt-HJ has stronger binding with tumor cells, better enhanced permeability and retention (EPR) effect, improved nuclease resistance, and less renal clearance.

In this study, Apt-HJ improved the antitumor efficacy in CT26-bearing mice vs. free PD-L1 aptamers (Figure 8A). The mechanisms may involve several aspects (Figure 9). First, HJ has four open ends that can connect with four aptamers to form the tetravalent Apt-HJ, which has a higher affinity to target tumor cells vs. the monovalent free aptamers (Figure 5, Figure 6 and Figure 7). Second, Apt-HJ has an average size of 13.22 nm, which is above the renal clearance threshold. This can prevent most aptamer loss through the kidneys, thereby prolonging the circulating time. Third, the 13.22 nm size of Apt-HJ is small enough to evade capture by the reticuloendothelial system (RES), and yet appropriate for Apt-HJ accumulation in tumor tissue via EPR effect. Fourth, the phosphorothioate modification of Apt-HJ can also prolong its circulating time. All these factors may have contributed to the enhanced in vivo antitumor efficacy of Apt-HJ vs. free PD-L1 aptamers. It should be noted that Apt-HJ was formed through self-assembly from four ssDNA chains, which might also form some intermediate products during the process. The mixture of the products needs to be purified if Apt-HJ is developed in the future for prospective clinical application.

In summary, ample evidence has shown that blocking PD-1/PD-L1 pathways can reduce immunosuppression and enhance antitumor immunity. In this study, a novel DNA nanostructure (Apt-HJ) comprising four PD-L1 aptamers and a HJ is constructed. The tetravalent Apt-HJ has higher affinity for tumor cells and improves the antitumor efficacy in vivo. Such multivalent DNA complexes may become a new strategy to further enhance the potential of PD-L1 aptamers in ICB applications.

## 4. Materials and Methods

### 4.1. Cells and Cultures

CT26 (mouse colon cancer cell lines) was obtained from the Cell Center of Chinese Academy of Medical Science (Beijing, China). The cells were cultured in DMEM medium supplemented with 10% fetal bovine serum (FBS) and antibiotics (100 U/mL penicillin and 100 μg/mL streptomycin). All cells were incubated in a 37 °C incubator with 5% CO_2_. DMEM was purchased from the Cell Center of Chinese Academy of Medical Sciences (Beijing, China). FBS was purchased from Biond (Kibbutz Beit-Haemek, Israel).

### 4.2. Synthesis of PD-L1 Aptamer

All DNAs were synthesized by Invitrogen (Shanghai, China). The four DNA strands for Apt-HJ had the following sequences, with the underlined part indicating the sequence of PD-L1 aptamer:

5′-CGGCGATCCGGCCATAGTGGATTGCGGGCCAGTGAAAAAAACGGGCCACATCAACTCATTGATAGACAATGCGTCCACTGCCCGT-3′;

5′-CTCACTGGCCCGCAATCCTGAGCACGTGGCTGACGAAAAAACGGGCCACATCAACTCATTGATAGACAATGCGTCCACTGCCCGT-3′;

5′-CCGTCAGCCACGTGCTCACCGAATGCTGCGCAACCAAAAAACGGGCCACATCAACTCATTGATAGACAATGCGTCCACTGCCCGT-3′;

5′-CGGTTGCGCAGCATTCGGACTATGGCCGGATCGCCAAAAAACGGGCCACATCAACTCATTGATAGACAATGCGTCCACTGCCCGT-3′.

The first nt at the 5′ end of each ssDNA for some Apt-HJ and all polyA-HJ was phosphorothioate modified.

### 4.3. Preparation of Apt-HJ

Four single-stranded DNA chains were dissolved in a saline solution (0.9% NaCl, 10 mM MgCl_2_). The concentration of each ssDNA was equal to 4 μM. The mixture was incubated at 95 °C for 5 min and slowly cooled to room temperature at a controlled rate of 0.1 °C/s.

### 4.4. Characterization of Apt-HJ

The prepared Apt-HJs were verified by using gel electrophoresis. The gel was made by dissolving 2.5% (*w*/*v*) agarose and GelStain (TransGen Biotech, Beijing, China) in TBE buffer. Samples were loaded onto the gel and run for 30 min at 120 V, and analyzed under UV light. The particle size and zeta potential of the Apt-HJ were evaluated by dynamic light scattering (DLS) using Zeta Sizer Nano ZS90 (Malvern Instruments, Malvern, UK) at 25 °C.

### 4.5. Serum Stability of Apt-HJ

The stability of the Apt-HJ in serum was also evaluated by agarose gel electrophoresis. Phosphorothioate-modified or non-modified Apt-HJs (4 μM) were incubated in 50% fetal bovine serum (FBS) at 37 °C. At the predetermined time points, the samples were heated immediately at 95 °C for 5 min to inactivate nucleases. Thereafter, electrophoresis was utilized to analyze the degree of Apt-HJ degradation. The gray-scale value was analyzed by Quantity One software (Bio-Rad Laboratories, CA, USA).

### 4.6. Evaluation of Cellular Binding Capacity

CT26 cells in a logarithmic growth phase were incubated in PBS (plus 0.02% EDTA) for 30 s and washed gently twice with PBS. The cells (2 × 10^5^) were suspended in 250 μL PBS. Next, 60 pmol FAM-labeled poly-A, Apt, or Apt-HJ were added to the cells, which were shaken gently for 30 min at room temperature. The cells were washed thrice with PBS and resuspended in 250 μL PBS. The samples were analyzed directly by Accuri C6 Flow Cytometer (BD Biosciences, San Jose, CA, USA). To evaluate the equilibrium dissociation constants (K_d_), CT26 cells (2 × 10^5^) were suspended in 250 μL PBS. Next, FAM-labeled Apt, or Apt-HJ were added to the cells. The final concentrations of Apt were 50, 100, 150, 200, 300, 600, 800, and 1000 nM, respectively. The final concentrations of Apt-HJ were 50, 100, 150, 300, 400, 600, and 1000 nM, respectively. Samples were shaken gently for 30 min at room temperature. The cells were washed thrice with PBS, resuspended in 250 μL PBS, and analyzed by flow cytometry (Accuri C6, BD, San Jose, CA, USA). The K_d_ of PD-L1 aptamers and Apt-HJ was determined by the relationship between the fluorescence intensity and the corresponding concentration [38,39] using the following equation: Y = Bmax X/(K_d_ + X).

### 4.7. Confocal Imaging Studies

CT26 cells were cultivated in a Lab-Tek Chamber #1.0 Borosilicate Coverglass System (ThermoFisher, Waltham, MA, USA) at a density of 1.5 × 10^4^ cells per well for 12 h. Subsequently, 100 pmol FAM-labeled poly-A, Apt, or Apt-HJ in 250 μL DMEM were added to the wells. Cells were further cultured for 4 h and washed three times with PBS. Paraformaldehyde solution (4%) freshly prepared and pre-cooled was applied for 10 min at 4 °C to fix the cells. The cells were washed three times with PBS and stained by Hoechst (Solarbio, Beijing, China) for 30 min at room temperature. Finally, cells were washed three times with PBS and incubated with 100 μL PBS. The fluorescence image was analyzed by a confocal laser-scanning microscope (Perkin Elmer Ultraview, Perkin, Waltham, MA, USA).

### 4.8. In Vivo Antitumor Study

Animal study and procedures were approved by the Ethics Committee of Institute of Basic Medical Sciences, Chinese Academy of Medical Sciences, according to the institutional animal care and use guidelines. BALB/c mice of 6-8 w old, with an average weight of 20 g, were purchased from Charles River Corp. (Beijing, China). To establish a colon cancer model, 5 × 10^5^ CT26 cells suspended in 100 μL PBS were injected subcutaneously on the right lower back. After tumor reached to a size of about 5 mm, mice were randomly divided into five treatment groups. The mice were treated with PBS, poly-A, poly-A-HJ, free-Apt, or Apt-HJ via intraperitoneal injection every two days for a total of six injections. The dosage of free PD-L1 aptamers was 1.2 mg/kg. Poly-A treatment had the same molar concentration as the PD-L1 aptamers. Since Apt-HJ and poly-A-HJ were tetravalent, the Apt-HJ or poly-A-HJ treatments were given a molar concentration that was 1/4 of the free PD-L1 aptamers. Tumor size and body weight were measured every 2 days during the experiment. Tumor volume was calculated according to the formula (a × b^2^)/2, where a and b represent the length and width of the tumor, respectively.

### 4.9. Statistical Analysis

Statistical analyses were performed by GraphPad Prism 8 software. One-way ANOVA with Fisher’s least significant difference (LSD) comparison test was used for statistical calculation. A statistically significant difference was defined as *p* < 0.05. Relevant data were presented as mean value and standard deviation.

## Data Availability

The data presented in this study are available in this article.

## Supplementary Materials

The following are available online, Table S1: Tumor volume measurements for the Apt and the Apt-HJ treatment groups.

## Sample Availability

Samples of the compounds are not available from the authors.
