# Supplementary material for: Novel Complex of PD-L1 Aptamer and Holliday Junction Enhances Antitumor Efficacy in Vivo
Source: Molecules. 2021 Feb 18;26(4):1067. doi: 10.3390/molecules26041067 (PMC7921949; doi:10.3390/molecules26041067)
Supplement: Supplementary file 1 [file molecules-26-01067-s001.pdf]

Table S1. Tumor volume measurements for the Apt and the Apt-HJ treatment groups

Apt	Day 7	Day 9	Day 11	Day 13	Day 15	Day 17	Day 19
Animal 1	7.47	9.40	20.00	32.48	38.58	95.37	142.19
Animal 2	4.58	11.44	37.58	40.08	65.24	98.22	159.07
Animal 3	0.50	0.86	12.36	22.23	51.59	141.82	262.26
Animal 4	0.50	4.74	14.23	44.85	104.74	171.90	330.46
Animal 5	11.80	19.35	33.69	51.28	81.97	164.80	200.32
Animal 6	7.75	10.47	33.65	37.65	93.87	194.02	301.43
Animal 7	12.91	15.92	27.63	53.03	107.41	192.19	218.13

Apt-HJ	Day 7	Day 9	Day 11	Day 13	Day 15	Day 17	Day 19
Animal 1	0.50	1.69	3.47	11.63	63.98	71.63	111.60
Animal 2	5.83	9.54	18.56	40.99	56.04	93.06	145.63
Animal 3	2.97	4.37	14.70	19.79	53.72	101.94	174.82
Animal 4	0.50	0.50	0.50	4.29	17.86	20.66	75.74
Animal 5	3.91	6.57	8.35	34.85	68.12	136.76	189.30
Animal 6	7.68	18.52	19.74	34.10	73.32	121.00	206.77
Animal 7	0.50	0.73	1.69	3.08	7.05	16.65	25.08
